# Tradeoffs and synergy between material cycles and greenhouse gas emissions: Opportunities in a rapidly growing housing stock

**DOI:** 10.1111/jiec.13576

**Published:** 2024-10-28

**Authors:** Sophia Igdalov, Tomer Fishman, Vered Blass

**Affiliations:** ^1^ Porter School of the Environment and Earth Sciences Tel‐Aviv University Tel Aviv Israel; ^2^ Institute of Environmental Sciences (CML) Leiden University Leiden The Netherlands

**Keywords:** circular economy, dynamic material flows and stocks, material use, residential buildings, scenarios, societal metabolism

## Abstract

Management of building materials’ stocks and flows is a major opportunity for circularity and de‐carbonization. We examine the relationship between material consumption and associated greenhouse gas (GHG) emissions under different scenarios in Israel, a developed country with an already high population density that expects tremendous growth in its housing stock by 2050. We created scenarios of varying housing unit sizes and additional material efficiency practices: fabrication yield, lifetime extension, material substitution, recycling, and their combination, resulting in 18 scenarios. In each scenario, the material flows and stocks needed to supply the housing demand and the resulting life‐cycle GHG emissions are quantified. No single material efficiency practice achieves a reduction in all indicators, suggesting a potential conflict between circular economy and decarbonization policies: The material substitution scenario allows for the biggest reduction in material consumption (12%–40% concrete reduction and 15%–51% steel reduction in 2050 compared with the baseline), while the recycling scenario achieves the biggest reduction in GHG emissions (22%–43% reduction in 2050 compared with the baseline). In the long‐term, the life‐extension scenario reduces most demolition waste. These findings can help policymakers and stakeholders consider the impacts of raw materials consumption and implement this knowledge in light of their priorities in policy packages. The results suggest a narrow window of opportunity within the next decade to influence material consumption and emissions to 2050. The findings could also shed light on the sustainability trajectories of other countries with similarly rapidly developing building stock, which have received little attention in this field.

## 1 INTRODUCTION

The building sector is among the most greenhouse gas (GHG) emissions‐intensive sectors, attributable to both the production of building materials and operational energy consumption (IEA, 2020). Materials production is responsible for 23% of total GHG emissions worldwide, over half of which is from steel and cement, lime, and plaster production (IRP, 2020). Steel and concrete are the most resource and GHG‐emission‐intensive materials used in buildings (Göswein et al., 2018; Lausselet et al., 2020). In 2020, households’ share of final energy consumption in the EU was 27% (Eurostat, 2023), and buildings represented 35% of energy‐related EU GHG emissions (European Environmental Agency, 2022). In the United States, residential energy accounts for about 20% of GHG emissions (Goldstein et al., 2020). In Israel, cement production alone was responsible for 54% of the GHG emissions generated by local industry in 2017 and for 4% of total country emissions (Ministry of Environmental Protection, 2021a), while housing represented 29% of electricity consumption in Israel and 14% of GHG emissions (Ministry of Environmental Protection, 2018). Countries have therefore established targets for more energy‐efficient and emissions‐efficient houses, which require a focus on the efficiency of construction materials (Annunziata et al., 2013; Carbon Neutral Cities Alliance, 2021; European Parliament, 2010).

Despite the importance of emissions from material production and consumption, in the buildings sector, most current policies still focus on use‐phase energy efficiency for GHG emissions reduction (Scott et al., 2018). However, material efficiency strategies can reduce 35%–60% of life cycle emissions from homes (IRP, 2020). To achieve circularity and de‐carbonization, a set of policy measures bundled as a policy package might be needed.

In this regard, Israel makes an interesting case study. Israel is a high‐income developed member of the OECD with one of the highest population densities in the world. Its building sector has become increasingly important because of a required growth in the number of housing units in upcoming decades, caused by the present deficit and the rapid surge in population (The National Economic Council, 2016). The stock of housing units in Israel has been growing rapidly from 2.1 million in 2012 to 2.5 million units in 2020 (CBS, 2022). According to the governmental forecast (The National Economic Council, 2016) and our extension of it to 2050 (SI.1.4), this stock is expected to grow by nearly 2 million units in the next 30 years, through growth rates ranging from 55,000 new units per year in 2021 to 67,500 new units per year from 2040 onward.

In Israel, 65–70 million tonnes of natural resources, mainly aggregates, are annually extracted for the building and infrastructure sectors (Ministry of Energy, 2021). Such intensive use of materials leads to significant threats to the economy and the environment (Bibas et al., 2021). Therefore, the government initiated an “Israel Resource Efficiency Center,” to provide information and support the transition to a circular economy (IREC, 2021).

During COP26, Israel pledged an ambitious commitment to zero net emissions by 2050 (Avis, 2021). However, the calculation of emissions from the building and construction material sectors includes only direct emissions from electricity consumption and local cement production, omitting embodied emissions or material efficiency strategies for reducing construction GHG emissions. Nevertheless, the adoption of a materials efficiency‐focused approach could increase recycling, reduce raw material consumption, and promote a faster transition to a low GHG‐built environment (Pomponi & Moncaster, 2016). The lack of data on future material demands in Israel hampers such advancements. Though some Israeli policy papers, for example, Ministry of Energy (2017), attempted to predict the consumption of aggregates based on housing unit growth rates, such reports dealt only with raw materials extracted within Israel and not the total amount of materials needed in the building sector. A recent estimation of the current and future consumption of gravel, sand, and cement in the building and infrastructure sectors concluded that Israel might face difficulties domestically supplying future demands for construction materials as early as 2025 (Zafuf, 2021).

Scenarios to illustrate different potential futures are frequent tools in climate change research and assessment. Scenarios provide a common basis for comparing different policies, impacts, and other action methods by assessing their influence on climate change (Riahi et al., 2017). A growing number of studies utilize material flow analysis (MFA) to assess future material cycles and their sustainability challenges under various scenarios. In the domain of nations’ buildings stock and its material flows, Müller's study of the Netherlands (2006) is considered the quintessential study utilizing prospective dynamic MFA with multiple future scenarios. This was followed by scenarios of the Chinese and Norwegian building stocks (Bergsdal et al., 2007; Hu et al., 2010; Huang et al., 2013), and further examples can be found in recent review articles (Fu et al., 2021; Lanau et al., 2019). This approach can be expanded to assess building materials’ lifecycle GHG emissions vis‐à‐vis resource efficiency (RE) and circular economy strategies. Pauliuk et al. (2021) and Zhong et al. (2021) did so on the scale of global regions. On the country scale, Germany (Pauliuk & Heeren, 2021), the United States (Arehart et al., 2022), and China (Song et al., 2023) are recent examples. They show that the potentials of resource efficiency strategies vary by country context and are difficult to generalize from one country's findings to another.

To the best of our knowledge, there has been no dynamic MFA study of materials in the building sector in Israel, and other types of assessments for Israel so far have not considered the impacts or potentials of material efficiency strategies on materials consumption and emissions. In this study, we focus on the quantification of the materials that would be needed to supply the housing demand in Israel by 2050 using dynamic MFA combined with GHG life cycle emissions data, under three socioeconomic scenarios and with varying levels of implementation of RE measures. The period to 2050 reflects the Paris Agreement's 2050 goals of reducing GHG emissions. Our objective is to assess the potential of RE strategies to lower material flows and emissions while fulfilling the expected growth in demands for housing and to identify whether there are win‐win scenarios in which both materials and emissions are reduced.

## 2 METHODOLOGY AND DATA

### 2.1 Framework

**FIGURE 1:**
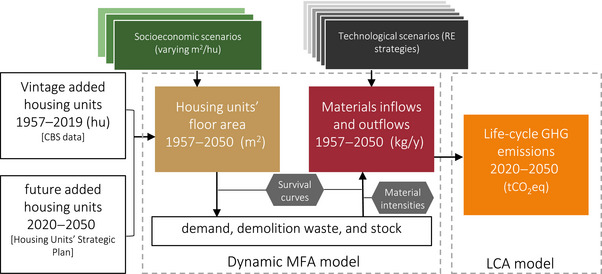
The model workflow. Socioeconomic scenarios correspond to different sizes of housing units, and technological scenarios represent the implementation of different resource efficiency strategies (buildings lifetime extension, materials substitution, etc.). MFA, material flow analysis; LCA, life cycle assessment; RE, resource efficiency; GHG, greenhouse gas.

We use a framework of future scenarios modeled with dynamic MFA and life cycle assessment (LCA), conceptually similar to other recent case studies (Barkhausen et al., 2023; Saadé et al., 2022). Our scenarios describe the fulfillment of future demand for housing units under various combinations of potential socioeconomic and technological developments in the Israeli residential sector. The turnover of housing units and the corresponding construction materials flows and stocks under each scenario are quantified with dynamic MFA. The materials’ life cycle GHG emissions are then calculated (Figure 1).

### 2.2 Model description

#### 2.2.1 Material flow calculation using dynamic MFA

We use a dynamic MFA model setup to calculate the accumulated stock and flows of housing units and corresponding construction materials. The MFA system boundaries include all dwellings in Israel from 1957 to 2050. The system quantifies the inflows, stocks, and outflows of steel, concrete, and cross‐laminated timber (CLT). Inflows are differentiated into primary virgin materials and secondary materials recycled from the outflows. Renovation processes are assumed to differ relatively little between the different scenarios, and due to the focus of this paper on the phases directly affected by the scenarios, the system does not include building renovation processes and their material flows.

The calculation was done by daisy‐chaining dynamic MFA models of housing units and their materials, as in Müller (2006) and the following studies: a flow‐driven model for the historical stock and outflows of housing units, which is extended by a stock‐driven model for the future housing units’ inflows and outflows. Then, these results feed a flow‐driven model to quantify the corresponding mass flows and stocks of construction materials. The dynamic MFA modeling and its equations and information flow are described in detail in the SI section 1.7.

#### 2.2.2 GHG emissions calculation using LCA

We calculated GHG emissions using the MFA results and emission intensity factors. These factors were adjusted to the appropriate materials and scenarios in cases they affected GHG emissions intensity, considering the functional unit and system boundaries. This assessment compares the different scenarios in terms of GHG emissions of the construction materials, not of the buildings. Thus, the LCA system boundaries were defined as cradle‐to‐grave including only stages that involve the use of different materials and do not include emissions related to the use phase of the building itself, such as in‐use energy consumption (light, air conditioning, etc.)

The functional unit was defined as the total gross floor space (m^2^) of the addition to the dwelling stock in Israel in year t per material. In the recycling scenarios, emissions and credits are included in the production process and not as part of the end‐of‐life treatment. That is, the impact of recycling resulting from outflows in a year was considered in the next year, as part of the production of the materials for that next year's required amount. For example, the emissions from the steel recycling process in year t were not included in year t but were considered in year t+1 as part of the production and use of recycled steel instead of virgin steel.

### 2.3 Scenarios

The required number of housing units from 2020 to 2050 is predefined across our scenarios by the government's Housing Units’ Strategic Plan (The National Economic Council, 2016). Our scenarios therefore compare the implications of different RE strategies to fulfill this predetermined stock of housing units, with the strategies applied separately or in combination with each other.

The scenarios are defined through three underlying storylines: Scenario S1 represents a freeze of current socioeconomic, technological, and policy trends in the Israeli housing sector; an intermediate S2 scenario with moderate advances toward sustainability; and scenario S3 exemplifies the most sustainable future by today's practical standards. The distinct storylines manifest as sets of input variables to the model, as detailed in Table 1. The three scenarios are fundamentally translated to housing sizes (floor space per housing unit) of new construction in the years 2020–2050 (See SI 1.1 for details). In S1, housing size is fixed at 183 m^2^/unit, the average historical size of 2009–2019. In S2, the average new housing unit gradually decreases to 129 m^2^/unit by 2050, equivalent to 36.8 m^2^ per person in 2050. S3 uses the target of 30 m^2^ per person by 2050 outlined in the low‐energy‐demand scenario (Grubler et al., 2018) and similarly applied in other studies (Fishman et al., 2021; IRP, 2020; Pauliuk et al., 2021). Thus, S2 and S3 apply more intense building use strategies compared to S1.

**TABLE 1:** Technological scenarios (2050 targets).

Resource efficiency strategy	Description	Default value	Scenario S1	Scenario S2	Scenario S3
**Housing size**	Average m^2^ per housing unit in 2050	n/a	183	129	105
**Lifetime extension**	Extension of buildings' mean lifetime in years	50	75	75	100
**Fabrication yield**	Reduction of material needed for construction	No reduction	2%	5%	10%
**Maximum recycling**	Share of recycled material in inflows (see details in SI.1.2)	No recycling	Concrete: 10% Steel: 10%	Concrete: 30% Steel: 50%	Concrete: 50% Steel: 90%
**Material substitution**	Share of CLT buildings in new construction	0%	20%	50%	80%
**Combined**	Combination of all scenarios with the addition of CLT recycling	All of the above	All of the above	All of the above	All of the above

*Note*: See SI.1.2 for detailed descriptions of these values.

Abbreviation: CLT, cross laminated timber.

Beyond the socioeconomic building use intensity strategies that are embedded in the basic S1–S3 scenarios, four further technical RE strategies are applied to each of these three scenarios: lifetime extension, fabrication yield improvement, material substitution from reinforced concrete construction to CLT buildings, and maximum recycling potential. In this last strategy, recycled steel replaces virgin steel and recycled concrete replaces virgin aggregate in new concrete with additional cement to maintain its strength. The maximum values achieved by 2050 for each of these four strategies vary between S1, S2, and S3 according to their scenario storylines (Table 1), based on accepted values in the literature as detailed in SI.1.2. These are “what if” strategies, to explore how they could affect material cycles and GHG emissions. Their feasibilities or likelihoods compared to local regulations, market conditions, future availability of materials, etc., are out of scope but important topics that will require further research.

In four variations on the basic three socioeconomic scenarios, the technological strategies are each applied one‐at‐a‐time to compare their relative effectiveness. A fifth variation combines the four strategies to show the maximum potential of all strategies applied together. In summary, our scenario space is composed of 18 scenarios: 3 socioeconomic housing size scenarios (S1, S2, S3) × 6 technological variations (no further strategies, housing unit size + one of the four other strategies, and housing size + all other strategies).

### 2.4 Data sources

#### 2.4.1 Historic housing unit data

Two sources were available for housing unit inflow data: (1) construction starts in terms of floor space 1974–2019 but not continuous (CBS, 2016, 2020a), and (2) construction starts in terms of housing units (CBS, 2020b) with a continuous annual data series from 1957 to 2019. Since our models work with units of floor space (cf. SI 1.7), we filled the gaps in the non‐continuous floor space data, assuming that housing unit size (m^2^/hu) changed over time. We applied a linear regression on these data and then calculated housing unit sizes in each year in m^2^/hu based on the regression results and normalized to the housing unit data in 1974 from the construction starts data (CBS, 2020b). 1974 was chosen because it was the first year of data. We finally multiplied these results by the housing unit data (CBS, 2020b) to get a continuous time series of the inflows of floor space for the entire period. Years in which the original data already existed remained the same. We further detail this approach and compare it to alternatives in the supporting information (SI.1.3).

#### 2.4.2 Data on future housing units

The governmental housing unit forecast (The National Economic Council, 2016) sets goals for the planned annual construction of new housing units for 2020–2040. We assume that the 2040 goals continue until 2050. The data source is in 5‐year intervals, leading to a stepwise time series. Since such jumps are unrealistic and unsuitable for use with a stock‐driven model, we smoothed this time series with a log‐linear ordinary least squares (OLS) regression, ensuring that the first year of the future time series, 2020, remains the same, detailed in the supporting information (SI.1.4). These data equate to the net addition to stock, that is, the inflows for expansion only, without inflows for replacement of end‐of‐life stock. The cumulative sum of these data is equal to the future stock in terms of housing units. The conversion to stock in terms of floor space varies between scenarios, as described in the next section.

#### 2.4.3 Material intensities

Concrete and steel are the two main materials we examine, and CLT is further explored in several scenarios as a substitute. Since no material intensity data for Israeli buildings exist in the literature to the best of our knowledge, we compiled concrete and steel material intensity coefficients (kg/m^2^) from local industry. These data fit in the upper range (9th–10th decile) of the material intensity values in the literature (Heeren & Fishman, 2019). For CLT, values from previous studies are used since no such CLT buildings currently exist in Israel. The material intensity data and their sources are described in the supporting information (SI.1.5).

#### 2.4.4 Lifecycle inventory

GHG emission factors were compiled from multiple sources, including industry LCA and EPDs, LCA software databases, and academic LCA studies (EPA, 2016; Igdalov et al., 2021; Lausselet et al., 2020; Martínez‐Rocamora et al., 2016; Menon, 2021; Ministry of Environmental Protection, 2021b; Notteboom & Cariou, 2009; Pearlmutter et al., 2013; Sea‐Disrances.ORG, 2021; Stora Enso, 2020; The Norwegian EPD Foundation & Pretec Norge AS, 2021; Zabalza Bribián et al., 2011; Hanson Israel Ltd., 2020; Harris et al., 2018; Holcim, 2014; Hossain & Ng, 2019; Hossain et al., 2016; Hoxha et al., 2020; Ltd., 2020; McIntyre et al., 2009; Meron et al., 2020; Nesher Israel Cement Enterprises Ltd., 2014; One Click LCA, 2021a, 2021b, 2021c; Wu et al., 2014). The sources are detailed in the supporting information (SI.1.6), describing the life cycle inventory values, the assumptions (e.g., place of production, transport), and the variations between scenarios with and without recycling.

## 3 RESULTS

### 3.1 The flows and stocks of floor space and materials 1970–2050

**FIGURE 2:**
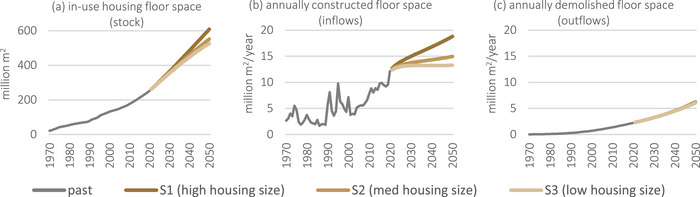
Historical and future total floor space of housing units in Israel: (a) in‐use stock, (b) construction inflows, and (c) demolition outflows. The future scenarios presented in this figure are the three base scenarios of different housing unit sizes. Note the vertical units and scale differ in (a) and (b) and (c). Since in the historical series and in these scenario variants material intensities per m^2^ do not change, these figures are identical in shape and proportion to the corresponding steel and concrete material flows and stocks. Underlying data are available in Supporting Information #2.

Israel's housing stock and accompanying flows of floor space have grown considerably in the last 50 years, as shown in Figure 2. Historical stocks grew by nearly 12 times from 21 million m^2^ in 1970 to over 250 million m^2^ in 2020, which equates to a growth of material stocks from 41 million tonnes to 480 million tonnes of concrete, and from 2 million tonnes to 24 million tonnes of steel. The historical stock growth rate has gradually increased due to surges in inflows in the 1990s and again in recent years. The frequent fluctuations of annual inflows in the 1990s do not manifest drastically in the stocks. Annually demolished stocks (outflows) were negligible in the early years (only 20,000 m^2^ consisting of 39,000 tonnes of concrete and 2,000 tonnes of steel) but dramatically grew by over a hundred times to 2.3 million m^2^, 4 million tonnes of concrete, and 214,000 tonnes of steel by 2020.

If the size of newly constructed housing units remains the same as in the last decade (i.e., scenario S1), the total floor space of housing in Israel will more than double in the next three decades (Figure 2a), requiring a continuous increase in annually constructed floor space (Figure 2b). However, by decreasing the floor space of new housing units, Figure 2b shows that annual floor space construction can grow much more moderately in S2 and even stabilize in S3, while still supplying the same number of housing units. Such actions would lead to reduced total in‐use floor space in 2050 by 9% (S2) to 14% (S3). The differences between the housing unit size scenarios do not substantially impact the demolished floor space by 2050 because most demolition is of floor space already constructed prior to 2020 (Figure 2c).

### 3.2 Demand for virgin materials in the resource efficiency scenarios

**FIGURE 3:**
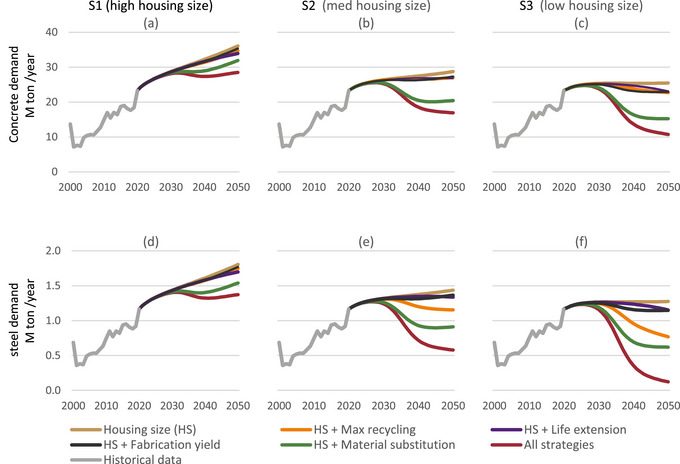
Demand (inflow) for virgin concrete (a–c) and steel (d–f) in the three scenarios and their six variants. The housing size variant (in brown) corresponds to the base scenarios in Figure 2b. Underlying data are available in Supporting Information #2.

Reducing the new housing size (scenarios S2 and S3), on its own, already leads to quite effective reductions of concrete and steel inflow demands. This can be seen in Figure 3b,e of scenario S2's brown series, which reaches similar values to those achieved by the employment of all other strategies together in S1, visualized as the red series in Figure 3a,d. In S3, the big reduction of housing size on its own stabilizes material demand, even without implementing any additional RE.

The materials substitution scenario from reinforced concrete to CLT construction, represented by the green line in Figure 3, is the most significant reducer of material demand of all the technical strategies when implemented one‐at‐a‐time (excluding the combination scenario). However, its impact varies between the housing unit scenarios. For S1, material substitution is relatively effective in reducing demand for steel and concrete, but it only temporarily stabilizes before increasing again. In contrast, for S3, the substitution scenario lowers materials demand back to 2010 levels and keeps it stabilized while nevertheless supplying a much higher number of housing units.

The four strategies of lifetime extension, material substitution, fabrication yield, and maximum recycling only become significant from around 2030, despite starting their implementation in 2021. This is in contrast with the housing size strategy whose impacts are more immediate.

Overall, the relative effectiveness of the RE strategies to reduce virgin inflow demand is maintained between the three scenarios for both concrete and steel. However, the impact of both maximum recycling and material substitution is more pronounced for steel than for concrete because of steel's higher recycling potential. It is less effective for concrete because of the additional cement that is required to compensate for the deteriorated quality of recycled concrete, and maximum recycling of concrete increases the total (both virgin and recycled) overall demand for concrete compared to the housing size only scenario variant.

### 3.3 Demolition waste to landfill

**FIGURE 4:**
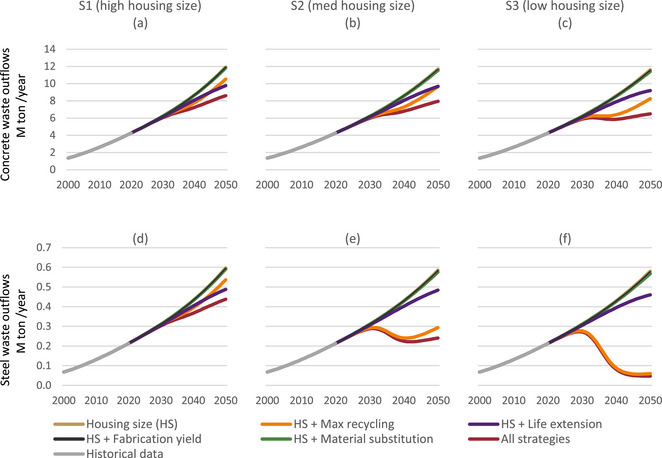
Demolition waste (outflow) of concrete (a–c) and steel (d–f) in the three scenarios and their six variants. The housing size variant (in brown) corresponds to the base scenarios in Figure 2c. Underlying data are available in Supporting Information #2.

Most demolition waste until 2050 is generated by the end‐of‐life of historical buildings already built prior to the implementation of any of the scenarios and strategies. Thus, most waste reduction potentials arise from improved recycling strategies. Figure 4 shows that on its own, reducing newly constructed housing sizes has negligible effect on demolition waste outflows of steel and concrete. Likewise, fabrication yield or material substitution has minimal impact on outflows of steel and concrete in all scenarios by 2050. This may change decades beyond the time horizon of 2050 when buildings with these strategies would reach their end of life.

In comparison, the maximum recycling and life extension scenarios lead to decreased demolition wastes to be landfilled prior to 2050. This decline is observed in both concrete and steel outflows in the high housing size scenario (S1, Figure 4a,d). In the medium (S2, Figure 4b) and low housing size scenarios (S3, Figure 4c), there is a reduction in concrete outflows and an absolute decrease in steel outflows (Figures 4e and 4f, respectively). However, there is a difference between the materials. In the case of concrete, maximum recycling achieves a higher reduction in the first decades, but the trends change and eventually, the life‐extension scenario becomes more significant (S2, Figure 4b) or is about to become so by 2050 (S3, Figure 4c). For steel, the maximum recycling scenario is the most significant one in scenarios S2 and S3.

The combination of all strategies while maintaining high housing sizes (S1) could potentially lower concrete and steel waste outflows by up to 28% by 2050. In contrast, incorporating reduced housing sizes alongside the technical strategies’ combination results in more pronounced reductions in waste outflows. Notably, only the more drastic housing size reduction in S3 can stabilize concrete outflows at around 6 million tonnes per year, commencing from around 2030. In this scenario, steel waste can be significantly curtailed by a factor of 10, reaching around 0.06 million tonnes per year by 2050.

The effects of the strategies only become pronounced after 2030. The maximum recycling strategy acts fastest to reduce outflows already between 2030 and 2040, but then outflows start increasing again, whereas life extension has a slower effect, which becomes more pronounced in later years.

### 3.4 GHG emissions

#### 3.4.1 Total annual GHG emissions

**FIGURE 5:**
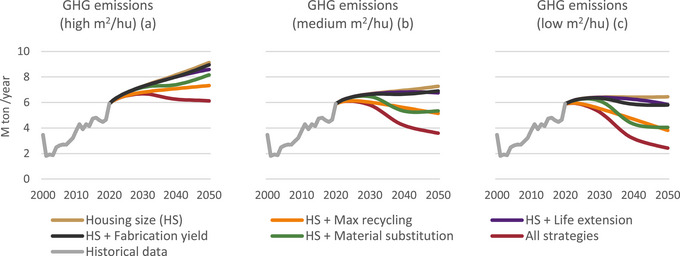
Annual greenhouse gas (GHG) emissions per technological scenario. Underlying data are available in Supporting Information #2.

Reducing the size of the housing units can stabilize the life cycle GHG emissions that occur as a result of the use of concrete and steel. While building housing units with the same average size as Israel does today (S1) will increase GHG emissions by 55% (Figure 5a), reducing their size will allow an increase of only 9% in 2050 compared to 2020 (Figure 5c). The recycling and material substitution scenarios drive the highest reductions. Also, it is interesting to note that the reaction times in the recycling and combination scenarios are shorter than in the other scenarios. In addition, in the long term to 2050, the max recycling scenario achieves better results compared with the substitution scenario. In the material substitution scenario, emissions increase in the last few years before 2050, though in the shorter term, this scenario shows greater reductions. The accumulated GHG emissions are described in the supporting information (SI3.1).

#### 3.4.2 Annual GHG emissions per material

**FIGURE 6:**
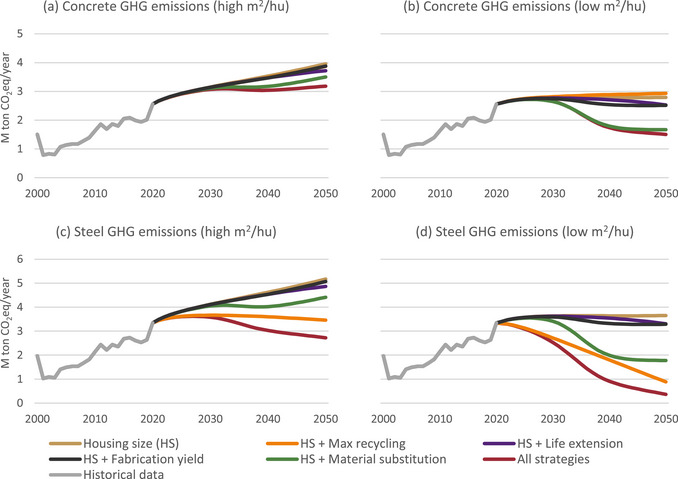
Annual greenhouse gas (GHG) emissions for concrete and steel per technological scenario. Underlying data are available in Supporting Information #2.

GHG emissions reduction from steel (Figure 6c,d) can be reduced more significantly than from concrete (Figure 6a,b) with the examined strategies. Especially, all the emissions reduction resulting from max recycling is related to the recycling of steel (76% in 2050 compared to the baseline housing size (HS) scenario, Figure 6d). For concrete, in scenario S3 (Figure 6b), the emissions even increase due to the increase in the demand for cement. In this case, the most efficient scenario is HS + material substitution, which allows a reduction of 13% in 2050 compared to the baseline HS scenario.

## 4 DISCUSSION

### 4.1 Material substitution reduces more GHG emissions

The material substitution scenario, in which CLT substitutes most concrete, was found to be the most efficient scenario in terms of GHG emission reduction, similar to other literature (Malmqvist et al., 2018). The CLT scenario might be even more significant in terms of GHG reduction if the CLT was recycled and reused. Because of the biogenic component, the recycling process has a significant GHG credit (Stora Enso, 2020).

Pursuing policies and actions to realize such a scenario can effectively support GHG emissions reduction. Nonetheless, it should be noted that our scenario does not consider the entire implementation process, which would be required because there are few CLT buildings in Israel today. Local CLT production is probably not feasible, though this is similar to most currently used construction materials other than local aggregates and cement. A market for imported CLT in mass construction would require new supply chains, know‐how, and bureaucratic means such as standardization, customs, and import and export laws. In addition, CLT is currently used for the construction of buildings up to 12 stories high. Its use in higher buildings, such as those nowadays commonly constructed in Israel, is still under development (Structural Timber Association, 2015).

### 4.2 Life extension reduces more waste outflows

In terms of material flows, the life‐extension scenario has the biggest effect on concrete waste reduction, which is in line with previous research (Bergsdal et al., 2007). In the literature, this measure was found to be insignificant for reducing GHG emissions in the middle term (Lausselet et al., 2020), since the impact of the life‐extension scenario on material consumption and GHG emissions would not be apparent for decades. Therefore, the target time horizon matters for assessing the potential of this strategy.

### 4.3 Circularity potential

**FIGURE 7:**
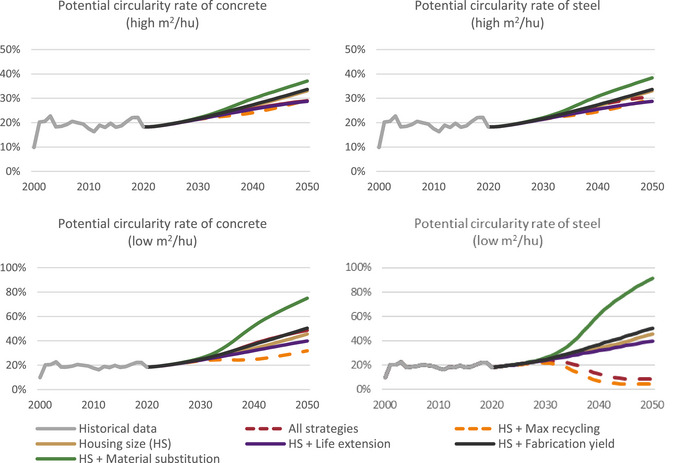
Potential circularity rate, calculated by the share of waste (outflow) divided by the demand (inflow) for concrete and steel in S3 (outflow/inflow). Recycling was already integrated into the max recycling and combination of all strategies scenarios, preventing direct comparison with others. Thus, these scenarios are marked with dashed lines. Underlying data are available in Supporting Information #2.

At present, the Israeli government promotes several plans to encourage a circular economy in the construction sector. Reuse and recycling are considered the main factors for this aim (Ministry of Economy & Industry, 2021). We could therefore ask, how much of the demands could be fulfilled by demolition waste in each scenario, disregarding technological limitations. This “circularity potential,” outflow divided by inflow, is seen in Figure 7. It has a low percentage in the max recycling scenario because, due to its definition, most of the waste had already been recycled. Overall, the circularity potential increases over the study period as a result of the increase in the generated waste, doubling between 2020 and 2050 in the baseline scenario. The highest circularity potential is in the substitution scenario because of its sharp decrease in the demand for concrete and steel. Our modeling does not consider the waste generated during the construction process, which would increase circularity potential. Furthermore, the past circularity potential rate of concrete does not capture actual historic recycling but rather past recycling potential that could have been realized.

Though this potential is high, its feasibility is challenging. As mentioned before, closed‐looped recycling of concrete is problematic. Steel recycling is technically possible, but currently, the local steel recycling industry is still limited. In the past, there were only metal separation and crushing activities. More recently, two metal recyclers have been operating. Yet their market share is still very limited, and the recycled materials are used for fences and other applications, and less for construction (CBS, 2023; CBS Energy, environment & agriculture unit, 2024). Rather, much of end‐of‐life steel is exported to Turkey for recycling, and it is more likely that in the future more steel with recycled content will be imported back to Israel, as their share increases.

### 4.4 Socioeconomic scenarios versus technological scenarios

The results of this study could be used to answer an interesting question: Which reduction technique has the greatest impact on material consumption and GHG emissions—a change in lifestyle using smaller housing units, or technology that enables material reduction? It should be noted that reducing the area per housing unit does not necessarily equate to lower wellbeing but can be achieved by a different lifestyle, for example, more common spaces, lower vacancy rates, less floor area for storage or vehicle parking, and so on. In Israel, such initiatives are still sporadic, such as private urban renewal initiatives that offer 20 m^2^ single‐person units (Globes, 2018) and governmental building regulations that allow—but do not yet require—reduction of in‐building private vehicle parking space under certain conditions (Ministry of Construction & Housing, 2016), so its overall feasibility remains to be explored further. Although literature on sufficiency related to building scenarios is still limited (Zell‐Ziegler et al., 2021), size reduction scenarios and other socio‐economic parameters can be framed around sufficiency (Gaspard et al., 2023). The results emerging from our scenarios can thus be further studied in the context of sufficiency and contribution to GHG emissions reductions from the residential buildings sector, by looking at the mechanisms and barriers to implementing such scenarios.

As long as housing sizes remain as they are (S1), no strategies reduce demand but only stabilize it (though still managing to provide the required housing units). In comparison, the demand for concrete in S3 in its baseline scenario is already lower than the demand for concrete in the most efficient scenario in S1. This shows that using technology alone is not enough; a change in our lifestyles must happen to achieve a significant reduction in materials and emissions. Combining the socioeconomic scenarios of size reduction with the technological scenarios leads to significant reductions in material demand (mainly using the substitution scenario) and GHG emissions (mainly using the recycling scenario). This is consistent with similar literature (Göswein et al., 2018; Pauliuk & Heeren, 2021; Zhong et al., 2021), though the numbers are unique to each case study.

### 4.5 No win‐win situation?

Our results suggest that there is no single strategy that achieves a simultaneous reduction in all target parameters for all materials. It rather varies between reduction of certain materials’ demand, reduction of demolition waste, or reduction of GHG reduction. For example, the maximum recycling scenario does not necessarily reduce GHG emissions, as can be seen in the case of concrete. Yet, for steel, this strategy aligns with both increased recycling and GHG reductions. This highlights that the circular economy is not necessarily always synonymous with climate change mitigation, corroborating findings from other regions and other economic sectors (Pauliuk et al., 2021; Song et al., 2023; Zhong et al., 2021). Accordingly, policy makers should set their goals and formulate policies based on their sustainability priorities. Nevertheless, the combination of the material substitution scenario and the max recycling scenario allows gains across the board. This combination would require the most sweeping changes from the current state, and realizing such a scenario would require possibly unprecedented collaboration between stakeholders in government, industry, and society.

The environmental impact of the max recycling scenario is complex: Although the max recycling scenario's effect on virgin material consumption is small compared with the other scenarios, its waste reduction efficiency is more significant in the case of steel. In comparison, for concrete, it is less effective over time than the life‐extension scenario. However, for the reduction of total GHG emissions, the max recycling scenario is the most efficient in the long term, due to its high recycling rate of steel (i.e., 100% in S3).

One reason for the low impact of the max recycling scenario on material demand can be explained by its position in the waste hierarchy (Gharfalkar et al., 2015). Recycling occupies the third position in the hierarchy, while prevention is the first step. The life‐extension and fabrication yield scenarios allow for reduction at the source. Therefore, in line with our results, recycling is a less preferable action in terms of material reduction.

### 4.6 A limited window of opportunity

By 2050, the total built area of dwellings is predicted to be 115%–150% greater than it is today. Depending on the technological environment, 40%–50% of the total GHG emissions we account for may have already occurred between 1970 and 2020, and the remaining 50%–60% spread over 2020–2050. Therefore, the impact of the housing sector on the environment is expected to be substantial in the next three decades. The strategies outlined in this study provide an opportunity to reduce this impact. However, the window of action to achieve this goal is limited.

It would take at least a decade before the implementation of strategies noticeably affects the inflows (demand) and outflows (waste) of materials. Hence, to achieve material reduction by 2050, the amount of time to act is less than 10 years. The right actions in the near future could stabilize the stock of construction materials. It requires the urgent promotion of policies and technologies that will allow the adoption of material efficiency strategies, as well as the development of recycling plants and the promotion of the importation of recycled materials. Delaying implementation strategies for more than a decade would lead to continuous growth in both demand and stock. In terms of GHG emission reduction, this window of action might be wider in the max recycling scenario because its impact was notable from the first year of its implementation.

### 4.7 Uncertainties

Modeling future pathways of material cycles involves large uncertainties and assumptions. By design, our what‐if scenario narratives describe the upper and lower bounds of extreme cases of growth trends and adoption (and lack of adoption) of technologies and strategies. In combination, the 18 scenarios provide a range of results that embody the inherent future uncertainty aspect, similar to other scenario‐informed studies that utilize dynamic MFA (Billy & Müller, 2023; Fishman et al., 2018; Pauliuk et al., 2021). As such, none of the scenarios are intended as forecasts or predictions, and there is no assumption of their relative likelihoods. There may also be uncertainties arising from the input data. The LCA data that we use are from verified sources, and uncertainty analysis is part of their performance protocol. Nevertheless, how well these types of data fit for future scenarios is subject to discussion, and future improvement of our model could apply prospective LCA approaches. We further address and discuss the sensitivity of assumptions that are debated in the literature, like the virgin concrete content in recycled concrete in SI section 1.2.3.

## 5 CONCLUSIONS

Our study shows that in a fast‐growing housing stock, no single strategy can succeed in reducing material demands, landfilled waste, and GHG emissions simultaneously. However, under a combined approach, all three sustainability targets can be reached while supplying the necessary growth of housing. Our findings highlight several next methodological steps. In the use phase, this study does not include the renovation, restoration, or replacement of building parts. Including these would provide a more comprehensive understanding of the total amounts of consumed materials and related GHG emissions during the entire lifetime of a building. Furthermore, massive dwelling construction requires additional infrastructure, such as roads, public buildings (e.g., schools), and so on. Therefore, the environmental impact of the government's housing unit strategic plan is greater than the impacts quantified in this study. Also, additional scenarios can be examined, such as repurposing offices or other non‐residential buildings to housing units that can reduce material demand without affecting housing unit size. Future research could determine the environmental impacts of housing unit plans using a consequential LCA approach. The expansion of system boundaries could accommodate additional material efficiency scenarios, such as downcycling aggregates for infrastructure (McIntyre et al., 2009).

Finally, as previously noted, the results of this research relate to technological implementation possibilities and limitations, and not to regulatory, standards, price markets, or other barriers. It is important to follow up with additional research that considers the overall barriers to the implementation of RE strategies. Moreover, some scenarios require more research and development to achieve their targets, such as ensuring a useful building lifetime of 100 years. One important general observation is that the interpretation of looking at GHG emissions is complex, especially where some of the materials like metals and CLT are produced abroad compared to cement, which is locally produced. Therefore, it is important to separate the local and global emissions in the future, as well as other potential environmental impacts like land use of more extensive wood use. This study forms a foundation for such follow‐up research.

## AUTHOR CONTRIBUTIONS

Sophia Igdalov and Tomer Fishman designed the study and the dynamic MFA model. Sophia Igdalov, Tomer Fishman, and Vered Blass formulated the scenarios. Sophia Igdalov collected the data, coded the model, and performed the computations with supervision from Tomer Fishman and Vered Blass. Sophia Igdalov and Vered Blass conducted the LCA. Sophia Igdalov, Tomer Fishman, and Vered Blass analyzed and drew conclusions from the results. Sophia Igdalov wrote the original draft. All authors contributed to the writing of the manuscript.

## CONFLICT OF INTEREST STATEMENT

The authors declare no conflicts of interest.

## Supporting information



Supporting Information #1 provides information on the housing unit size in each scenario, further details of the recycling values, different methods to fill historical gaps in the housing units’ data, data on predicted housing units—regression details, material intensity data, detailed lifecycle inventory, and the detailed dynamic MFA model description.

Supporting information #2 provides the data from all figures in the manuscript and SI1.

Supporting information #3 provides additional figures for this study.

## Data Availability

The data that supports the findings of this study are available in the supporting information of this article.
