# Supplementary material for: Tradeoffs and synergy between material cycles and greenhouse gas emissions: Opportunities in a rapidly growing housing stock
Source: J Ind Ecol. 2024 Oct 28;28(6):1912–25. doi: 10.1111/jiec.13576 (PMC11667649; doi:10.1111/jiec.13576)
Supplement: Supplementary file 1 — Supporting Information #1 provides information on the housing unit size in each scenario, further details of the recycling values, different methods to fill historical gaps in the housing units’ data, data on predicted housing units—regression details, material intensity data, detailed lifecycle inventory, and the detailed dynamic MFA model description. [file 44498_2024_2806042_MOESM1_ESM.pdf]

SUPPORTING INFORMATION FOR:

Igdalov, S., Fishman, T., Blass, V. (2024).
Tradeoffs and synergy between material cycles and greenhouse gas emissions: Opportunities in a rapidly growing housing stock. Journal of Industrial Ecology.

Summary

SUPPORTING INFORMATION FOR: 1
SI 1.1. Housing unit size in each scenario 2
SI 1.2. Technological strategies and scenarios 2
SI 1.3. Methods to fill historical gaps in the housing units data 4
SI 1.4. Data on predicted housing units – regression details 6
SI 1.5. Material intensity data 7
SI 1.6. Lifecycle inventory 8
SI 1.6.1. Scenarios without recycling 8
SI 1.6.2. Scenarios that include recycling 10
SI 1.7. Detailed dynamic MFA model description 12
References 13

SI 1.1. Housing unit size in each scenario

Figure SI1.1 shows the change in housing unit size in each scenario. A linear method was used to smooth historical data (CBS 2020a).

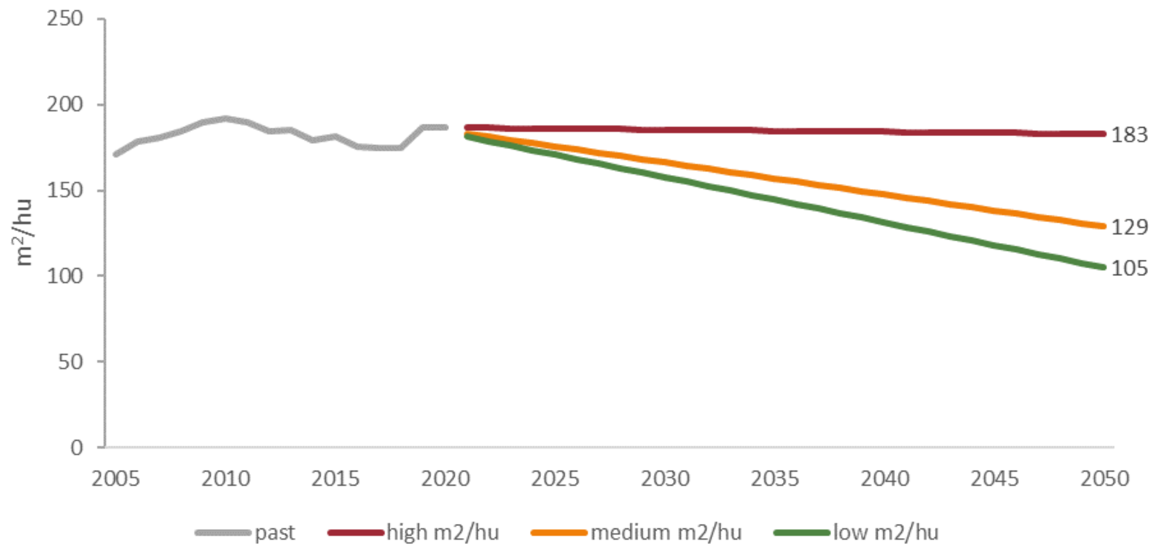

Figure SI1.1: Housing unit size per socioeconomic scenario

SI 1.2. Technological strategies and scenarios

Life extension

According to the literature, a building's lifetime ranges between 50 and 100 years (Cabeza et al. 2014). For this reason, a mean lifespan of 50 years was used for the Baseline scenario, 75 years was used as improvement in S1 and S2, and 100 years represented the upper limit of S3.

Fabrication yield improvement

This strategy focuses on material loss reduction during the construction phase. To attain material loss reduction, different steps can be taken, one of which is an advanced method of construction. This method allows for the production of the elements in a plan, which are combined on the construction site. According to the national plan for advanced construction methods, this allows to achieve a 10% reduction in the use of the materials (Ministry of Construction and Housing 2019). According to this limit, the targets for S1 and S2 were defined as 20% and 50%, respectively, of the maximum.

Maximum Recycling

The maximum recycling scenario concerns the rates of recycled material in the total amount of material used to build new buildings. Several assumptions on this were based on consultations with an expert (A. Katz, personal communication, 10 August 2021).

Steel is known to be a highly recycled material, and its maximum recycling rate ranges from 90%–93% (American Institute of Steel Construction 2021). It is important to mention that the characteristics of recycled steel remain the same as virgin steel. Hence, the potential recycling level of steel waste is equal to the potential amount of recycled steel that can be used for construction (Carbon Smart 2021). In S3, the recycling rate of steel was 90%. In S1, there was a low rate of 10%, and in SES2, the rate was 50%.

Concrete recycling is more complex. Concrete waste can be used to produce recycled aggregate, which can replace some virgin aggregates in concrete components. Today, the maximum rate of recycled aggregate that can be used is 20% of the total aggregate weight (Zhutovsky & Shishkin, 2020).

In the future, it is expected that 50% of recycled aggregate could be used. However, the addition of recycled aggregate changes the strength characteristics of concrete. A higher share of recycled aggregate, up from 20%, requires a higher share of cement to maintain the same strength (McIntyre et al., 2009; Zhutovsky & Shishkin, 2020). When we assess concrete with recycled aggregate in the maximum recycling strategy, the recycling rate refers to the percentage of recycled aggregate. In S1, the recycling rate that was used was 20% without a change in cement intensity. In S2, the recycling rate was assumed to be 30% reused concrete as aggregate, with a 10% increase in cement intensity to compensate for the loss in concrete strength. In S3, a recycling rate of 50% was assumed with an expansion of 20% in cement intensity.

Material substitution

An alternate material was cross-laminated timber (CLT), which is known as a low carbon material that is increasingly commonly used across the world. Information about this material appears in the sections based on a personal communication with an expert (D. Zohar, personal communication, August 2021).

CLT is a massive wood construction product consisting of at least three single-layer panels that are bonded together crosswise. It is currently used in place of concrete, masonry, and steel in the construction of commercial, industrial, and residential buildings. The optimal height of a CLT building is 6–8 stories, while CLT buildings can reach up to 20 stories.

CLT buildings are replacing concrete buildings, which are differentiated by various penetration rates. Based on the distribution of housing units according to the number of stories (CBS 2020b), in S3 it was assumed that 70% of housing units were CLT buildings; in S2, 50%; and in SES1, 20%.

1.2.5 All strategies scenario

The "all strategies" scenario combine all previous strategies with the addition of CLT recycling.

SI 1.3. Methods to fill historical gaps in the housing units data

Two methods were considered to fill the gaps of the historical added built floor area: a linear method and a two years moving average. In addition, a sensitivity check had provided for the normalization year that was used to calculate the vintage size of the housing units: 1974 and 1990.

Figure SI1.2 presents the original gross addition to stock (GAS) data and the linear regression equation that was used to fill the gaps in the data.

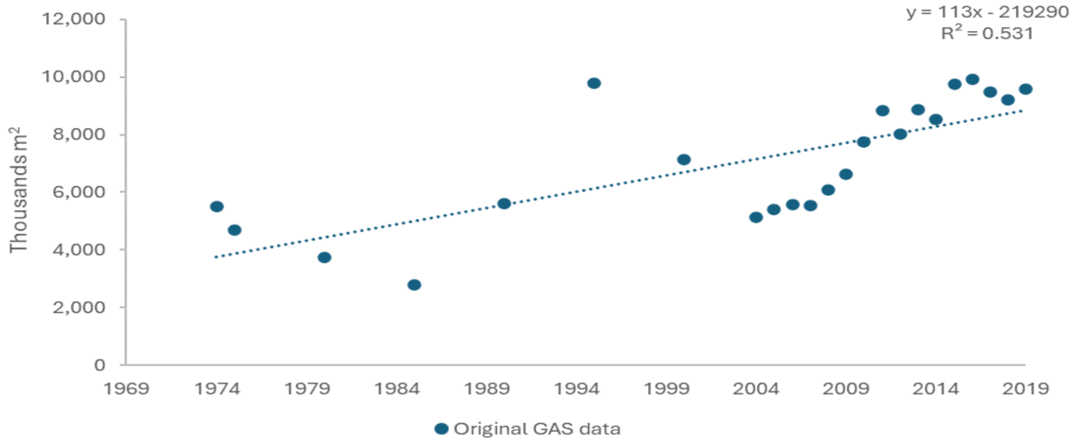

Figure SI1.2: Original GAS data and the linear regression to fill the gaps. The regression equation and R^2 are presented on the right side.

Figure SI1.3 presents the added m^2 area (i.e. GAS) per year, with each of the methods. The trends are similar though there are some differences in absolute numbers.

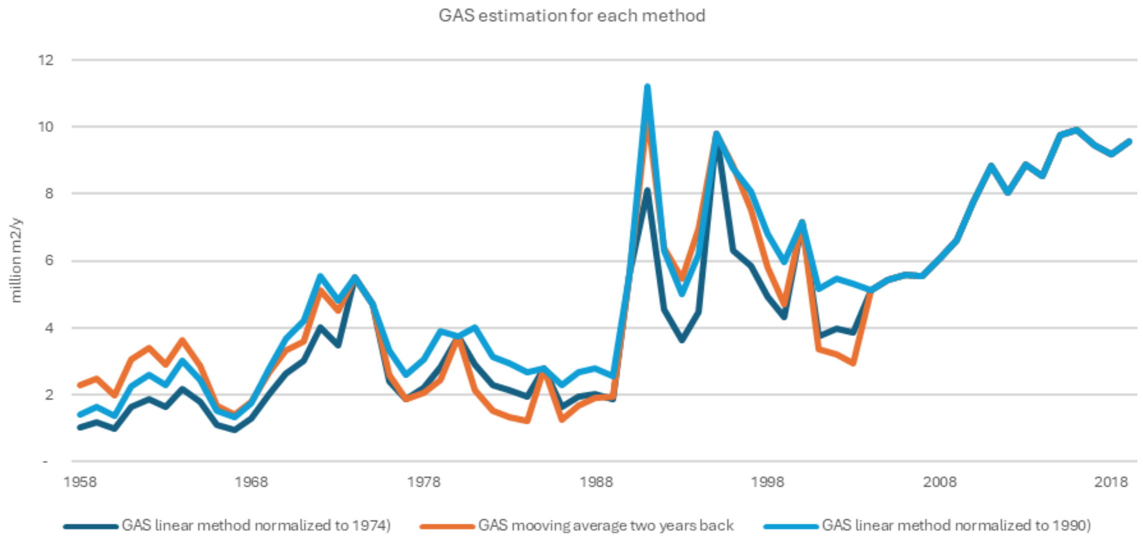

Figure SI1.3: Added m^2 area of dwellings per year (GAS), using different methods to fill the gaps in data.

Figure SI1.4 presents the results of the MFA model in terms of area (m^2) for demolition and stock of built area for each of the methods. Different GAS causes different absolute results in the stock (6%-14% addition) and demolition areas (20%-27% addition), though the trends are similar. The objective of this research is to compare the impact of different ME strategies on future consumption. Accordingly, as long as each method presents the same trend, the absolute numbers will not change the conclusions of this study, but only the absolute quantity of stock and outflows. It was therefore decided to use the linear method with normalization to 1974 to as a conservative option (it presents the lowest values) and for maintaining modeling simplicity.

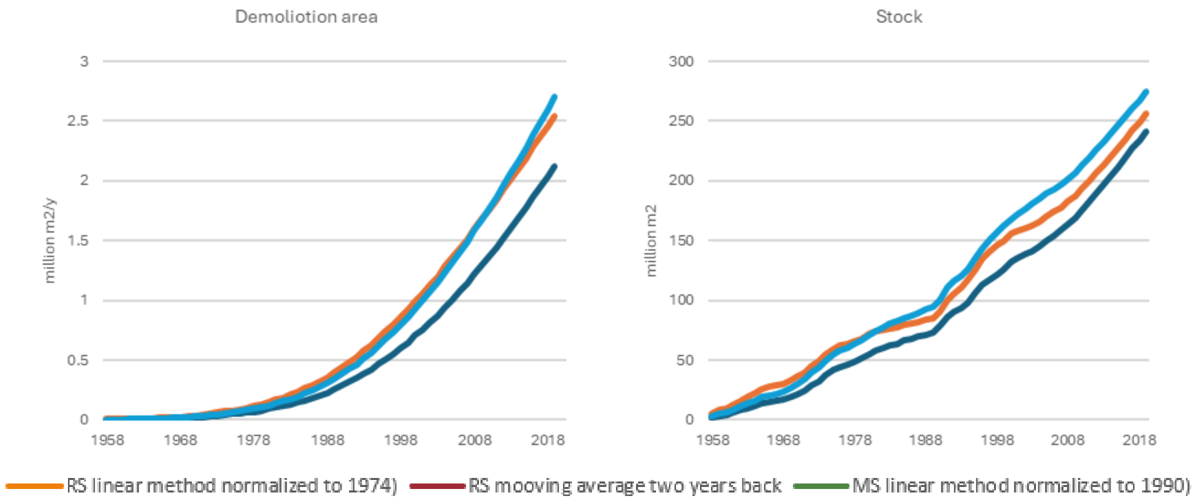

Figure SI1.4: RS and MS, using different methods to fill the gaps in data

SI 1.4. Data on predicted housing units – regression details

The data of the governmental housing units forecast (The National Economic Council 2016) includes goals for planned housing unit construction until 2040. We continued the goals from 2040 until 2050, assuming the same number of added housing units. Figure SI1.5 presents the number of housing units added each year. The forecast data is in 5-year intervals leading to a step-shaped inflow trend, which is inadequate as input to a stock-driven dynamic MFA model. To smooth the growth, a log-linear prediction (logarithmic function) was used, represented by the dashed line, while the original value in 2020 remained the same.

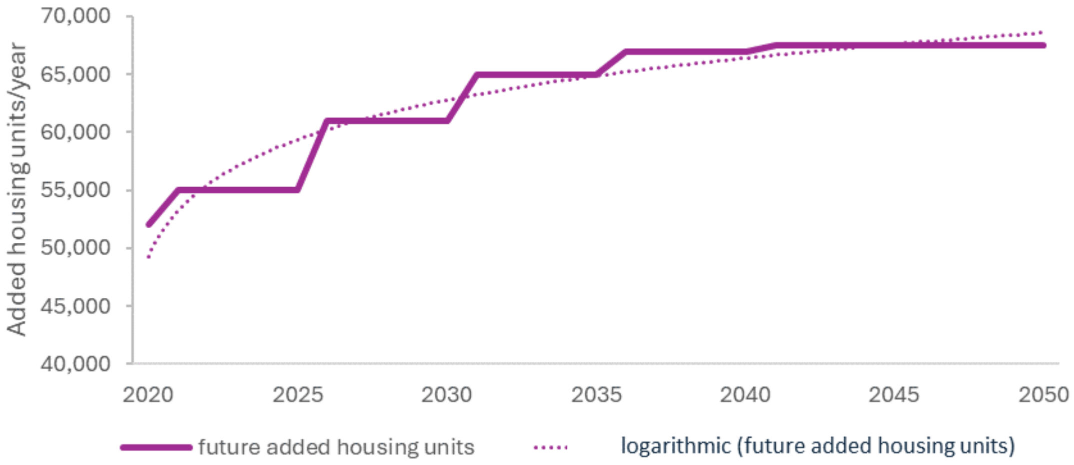

Figure SI1.5: Housing units added annually

The calculation of the added m^2 was based on the prediction of housing unit size. This prediction was made using a linear regression based on estimated past data and housing units' size according to the socioeconomic scenario. The prediction differs by scenario.

SI 1.5. Material intensity data

Table SI1.1 summarizes our material intensity data.

Table SI1.1: Material intensity data

BUILDING TYPE	***MATERIAL***	***MATERIAL INTENSITY (KG/M²)***	***DATA SOURCES***	***COMMENT***
Typical Israeli residential building	Concrete	1920	(Hanson Israel Ltd. 2020)	Assumed a density of 2.4 ton/m^3
	Steel	96	(Ashtrom Group Ltd. 2020)	Assumed 120 kg for one ton of concrete
CLT building	Concrete	813	(Durlinger et al. 2013; Jayalath et al. 2020)	Average of the two sources, as described in Table SI1.2.
	Steel	26		
	CLT	174		

CLT building material intensity is based on an average of two sources (kg/m^2), as presented in Table SI1.2: Material intensity in a CLT building.

Table SI1.2: Material intensity in a CLT building

MATERIAL	**(DURLINGER ET AL. 2013)** ***NOTE: THE VALUES ARE AN AVERAGE OF LOW AND THE HIGH CONSUMPTION PRESENTED IN THIS STUDY.***	**(JAYALATH ET AL. 2020)**	***AVERAGE (KG/M²)***
Concrete	801	825	**813**
Steel	2.86	48	**26**
CLT	253	96	**174**

SI 1.6. Lifecycle inventory

The GHG emission factors were separated between scenarios with and without recycling.

SI 1.6.1. Scenarios without recycling

Table SI1.3 presents the emission factors for each material without considering recycling.

Table SI1.3: Emission factors without recycling

MATERIAL	SYSTEM BOUNDARIES	VALUE (KG CO_2EQ/KG)	SOURCE
Steel	Cradle-to-site	2.87	(The Norwegian EPD Foundation and Pretec Norge AS 2021)
Concrete	Cradle-to-site	0.11	Various sources – see Table SI1. 5
CLT	Cradle-to-grave	0.38	(Stora Enso 2020)

As shown in Table SI1.4, the emission factors were in the same range as in previous studies. Specifically, the emission factor for concrete was lower compared with the other materials.

Table SI1.4: GHG emission factors in the literature

MATERIAL	EMISSIONS FACTOR (KG CO2EQ/KG)	SOURCE
Steel	1.03-3.19	(Martínez-Rocamora et al. 2016)
	0.77-3.75, 2.27 on average	(Lausselet et al. 2020)
	2.89	(One Click LCA 2021a)
Concrete	0.07-0.12	(Martínez-Rocamora et al. 2016)
	0.08-0.19, 0.12 on average	(Lausselet et al. 2020)
	0.14	(Zabalza Bribián et al. 2011)

Main assumptions for steel

- The main importation of steel to Israel is from Turkey. Because of the lack of data on emissions from steel production in Turkey, it was assumed that the Norway data, which is the most available source, represented steel production in Turkey.
- The transportation distance data were calculated separately:
    - Shipping from Istanbul to Haifa Port (stage A4)
        - Shipping distance: 1,843 km (Sea-Disrances.ORG 2021)

    - Average fuel consumption: 200 tons of fuel oil/day (assuming a speed of 23 knots and size 8,000 TEU) (Notteboom and Cariou 2009)
    - Average steel weight per containership: 24,000 kg/TEU (Menon 2021)
    - The GHG emission factor of fuel oil: 3.08 tons CO_2eq/ton fuel (Ministry of Environmental Protection 2021)
  - From Haifa Port to the construction site (stage C2)
    - Average distance: 99 km (Pearlmutter et al. 2013)
    - GHG emissions per km by truck: 237 gCO_2/km (Igdalov et al. 2021)
- Emissions from landfills were not included in the calculation because of their minimal impact (EPA 2016).

Main assumptions for concrete

- The emissions factor for concrete was calculated based on the emissions from the production of each component and the share of this component in concrete weight, based on the composition presented in Table SI1. 5 (Hanson Israel Ltd. 2020).

Table SI1. 5: Shares and emission factors of concrete components

MATERIAL	KG/M^2	SHARE	EMISSIONS FACTOR (KG CO_2EQ/KG)	SOURCE (EMISSION FACTORS)
Cement	250	11%	0.77	(Nesher Israel Cement Enterprises Ltd. 2014)
Natural aggregate (gravel)	1800	77%	0.03 / 0.003*	(Hossain and Ng 2019; Holcim 2014)
Water	300	13%	0.002	(Meron et al. 2020)

Because of the large differences in this emission factor in the literature, a sensitivity check was conducted using different values. The results in this sensitivity check are very similar to those presented as the main scenario. The lower emission factors cause lower emissions in all the scenarios. In the Combination scenario, the reduction compared to the main scenario is 11%, 10%, and 9% respectively with S1, S2, and S3 (the results are presented in SI3.7).

- Because of lack of information, data on emissions from end-of-life processes (stages C1–D) were not included. As a result, emissions from concrete were missing from the assessment. However, emissions from landfills had a minimal impact on the total emissions factor (Wu et al. 2014).

- The average transportation distances are shown in Table SI1.6. These distances represented a weighted average of transportation distance by region.

Table SI1.6: Local travel distances per material (Pearlmutter et al., 2013)

MATERIAL	DISTANCE (KM)
Concrete	11
Cement	45
Gravel	38

GHG emissions per km by truck: 237 gCO_2/km (Igdalov et al. 2021)

Main assumptions for CLT

- CLT density: 470 kg/m^3

As a result of plant growth, biogenic carbon accumulates in the biomass of plants. Previous studies in the literature disagree on whether this carbon should be considered a greenhouse gas emission because it is derived from a natural resource. Researchers who do not consider biogenic carbon usually do so according to the carbon neutrality assumption, which states that biogenic carbon sequestered during growth is released back to the atmosphere in the same amount and form by either naturally decomposing or burning so that atmospheric GHGs do not increase. However, several studies have questioned this approach because factors such as forestry management and the duration of temporary sequestration in buildings have not been considered (Harris et al. 2018; Hoxha et al. 2020). Therefore, as a conservative assumption, the results presented here include biogenic carbon. The results that do not include this component are presented in SI3.6. In this case, the emission factor used was 0.85 $kgCO_2$eq/kg CLT.

- The transportation distances (stage A4) representing EU average distances are not displayed.

SI 1.6.2. Scenarios that include recycling

Table SI1.7 presents the emission factors in the production and use of recycled materials. Note the recycling rates of each scenario considered. For example, in a scenario with 10% recycled material, the emission factor represents a weighted average of 10% recycled material and 90% virgin material.

Table SI1.7: Emission factors in recycling

MATERIAL	SYSTEM BOUNDARIES	SES1	SES2	SES3	SOURCE

Steel	Cradle-to-site	The calculation method is described in the following section			(One Click LCA 2021a, 2021b, 2021c)
	Share of recycled material	10%	50%	90%	
*Concrete**	Cradle-to-site	0.106 / 0.086	0.112 / 0.093	0.116 / 0.100	(Hossain et al. 2016; McIntyre et al. 2009)
	Share of recycled material	10%	30%	50%	

* Because of the large differences in this emission factor in the literature, a sensitivity check was conducted using different values each time. Excluding the biogenic component makes the difference between the Max Recycling scenario and the Substitution scenario bigger due to the higher emissions in the Material Substitution scenario. As a result, the reduction achieved by the Combination scenario is lower by 3%, 16%, or 29% in S1, S2, or S3 respectively (the results are presented in SI3.6).

Main assumptions for recycled steel emission factor

Because there are no steel recycling plants in Israel, it was assumed that recycling would take place overseas and imported steel would already contain recycled materials. Here, the emission factor did not include any emissions from end-of-life treatment; these emissions were assumed to have been included in the production of imported recycled materials. The emission factors were calculated based on data from OneClick LCA on reinforcement steel produced with 0%, 60%, and 90% recycled materials (One Click LCA 2021a, 2021b, 2021c). To calculate the emissions by 10% and 50% recycled material, a linear prediction was used with the emissions related to the production of virgin steel6. The rest of the assumptions remained the same.

6Main assumptions for recycled concrete emissions factor

- Same assumption as for virgin concrete.
- The data on recycled aggregates represented the production of recycled aggregates and not end-of-life treatment.

- The emission factors in the production of recycled aggregate were 0.012 (Hossain et al. 2016) and 0.0006 (McIntyre et al. 2009).

SI 1.7. Detailed dynamic MFA model description

Flow-driven model for the historical and current housing unit stock

We use a flow-driven dynamic MFA model to calculate the historical accumulation of in-use housing unit stocks:

$$Stock_{area}(t) = \sum_{\tau=t_0}^{t}[inflow_{area}(\tau) \times survival(t-\tau,\mu,\sigma)] \quad \text{Eq.1}$$

$Stock_{area}(t)$ is the total floor space (m^2) of housing units in time t from 1957 to the most recent year of available data, 2019. It is the sum of the surviving amount of each cohort of annual gross addition to housing unit stocks $inflow_{area}$ up to time t. The depreciation of each cohort over time, from 100% towards 0%, is described by $survival$. This survival curve follows the s-shaped complementary cumulative function of the normal distribution. This assumption is standard in the literature (Cabeza et al. 2014). The mean μ of the normal distribution varies by cohort and by scenario, and the standard deviation is $\sigma = \mu/3$ to maintain the proportional shape of the survival curve.

Stock-driven model for the inflows of future housing units

The second dynamic MFA model is a stock-driven model whose inputs are the historical stocks calculated in the previous stage and extended by the assumed future stock demand, and the survival curve. This model calculates the future additions of housing units $inflow_{area}$ in year t that will be required to fulfill the $Stock_{area}$ demand of that year, including the demand for replacement of end-of-life housing units from previous years' inflows due to their depreciation:

$$inflow_{area}(t) = \frac{Stock_{area}(t) - \sum_{\tau=t_0}^{t-1}[inflow_{area}(\tau) \times survival(t-\tau,\mu,\sigma)]}{survival(0,\mu,\sigma)} \quad \text{Eq.2}$$

Where future $Stock_{area}(t)$ and $survival(t - \tau, \mu, \sigma)$ vary according to the scenario, and $inflow_{area}$ cohorts up to year $t - 1$ on the right hand side are calculated recursively. τ is the index of previously added inflow cohorts.

Flow-driven model for the flows and stocks of construction materials

In this step, the material inflows and stocks of concrete, steel, and CLT (in kg) for the entire period are calculated with $material\ intensity$ coefficients (kg/m^2). The value of $material\ intensity$ can vary over time, which means that housing unit stocks can be composed of cohorts with different material intensities. Therefore, first the inflows of each material $inflow_{material}(t)$ are quantified by multiplying housing unit inflows $inflow_{area}$ (m^2) from the previous two models by their $material\ intensity$:

$$inflow_{material}(t) = inflow_{area}(t) \times material\ intensity_{material}(t) \quad \text{Eq.3}$$

Then the material stock $MS_{material}(t)$ was calculated separately for each material, using flow-driven dynamic MFA:

$$MS_{material}(t) = \sum_{\tau=t_0}^{t} [inflow_{material}(\tau) \times survival(t - \tau, \mu, \sigma)] \quad \text{Eq.4}$$

Then, outflows of each material in each year t were estimated using mass balance, since the outflow is equal to the inflows minus the net change in stock in two successive years:

$$outflow_{material}(t) = inflow_{material}(t) - \Delta MS_{material}(t, t-1) \quad \text{Eq.5}$$

References

American Institute of Steel Construction. 2021. Structural Steel Sustainability. https://www.aisc.org/why-steel/sustainability/. Accessed March 2, 2022.

Ashtrom Group Ltd. 2020. Personal communication. November 30.

Cabeza, L.F., L. Rincón, V. Vilariño, G. Pérez, and A. Castell. 2014. Life cycle assessment (LCA) and life cycle energy analysis (LCEA) of buildings and the building sector: A review. *Renewable and Sustainable Energy Reviews* 29: 394–416.

Carbon Smart. 2021. Carbon Impact of Steel. https://materialspalette.org/steel/.
CBS. 2020a. Construction – selected data (table 20.1). https://www.cbs.gov.il/he/publications/doclib/2019/20.%20shnatonconstruction/st20_01.xls.
CBS. 2020b. *Construction area by purpose and construction stage (table 20.6)*. August. https://www.cbs.gov.il/he/publications/doclib/2020/20.%20shnatonconstruction/st20_06x.xls.
Durlinger, B., E. Crossin, and J. Wong. 2013. Life Cycle Assessment of a cross laminated timber building. www.fwpa.com.au.
EPA. 2016. *Documentation for Greenhouse Gas Emission and Energy Factors Used in the Waste Reduction Model (WARM)*. https://www.epa.gov/sites/default/files/2016-03/documents/warm_v14_containers_packaging_non-durable_goods_materials.pdf.
Hanson Israel Ltd. 2020. Personal communication. September 11.
Harris, Z.M., S. Milner, and G. Taylor. 2018. Biogenic Carbon-Capture and Sequestration. *Greenhouse Gas Balances of Bioenergy Systems*: 55–76.
Holcim. 2014. *EPD of Aggregates*. Romania. www.environdec.com.
Hossain, M.U. and S.T. Ng. 2019. Influence of waste materials on buildings' life cycle environmental impacts: Adopting resource recovery principle. *Resources, Conservation and Recycling* 142: 10–23.
Hossain, M.U., C.S. Poon, I.M.C. Lo, and J.C.P. Cheng. 2016. Comparative environmental evaluation of aggregate production from recycled waste materials and virgin sources by LCA. *Resources, Conservation and Recycling* 109: 67–77.
Hoxha, E., A. Passer, M.R.M. Saade, D. Trigaux, A. Shuttleworth, F. Pittau, K. Allacker, and G. Habert. 2020. Biogenic carbon in buildings: a critical overview of LCA methods. *Buildings and Cities* 1(1): 504–524.
Igdalov, S., R. Kamara, A. Zaltzberg, A. Eshet, and Y. Laster. 2021. External environmental costs of road transport - air pollutants and greenhouse gases (עלויות חיצוניות סביבתיות של תחבורה כבישית - מזהמי אוויר וגזי חממה). *Ministry of Environmental Protection*.
Jayalath, A., S. Navaratnam, T. Ngo, P. Mendis, N. Hewson, and L. Aye. 2020. Life cycle performance of Cross Laminated Timber mid-rise residential buildings in Australia. *Energy and Buildings* 223: 110091.
Lausselet, C., J.P.F. Urrego, E. Resch, and H. Brattebø. 2020. Temporal analysis of the material flows and embodied greenhouse gas emissions of a neighborhood building stock. *Journal of Industrial Ecology*: 1–16.
Martínez-Rocamora, A., J. Solís-Guzmán, and M. Marrero. 2016. LCA databases focused on construction materials: A review. *Renewable and Sustainable Energy Reviews* 58: 565–573.
McIntyre, J., S. Spatari, and H.L. MacLean. 2009. Energy and Greenhouse Gas Emissions Trade-Offs of Recycled Concrete Aggregate Use in Nonstructural Concrete: A North American Case Study. *Journal of Infrastructure Systems* 15(4): 361–370.
Menon, H. 2021. TEU in Shipping - Everything You Wanted to Know. *Maritime Law*. https://www.marineinsight.com/maritime-law/teu-in-shipping-everything-you-wanted-to-know/.
Meron, N., V. Blass, and G. Thoma. 2020. A national-level LCA of a water supply system in a Mediterranean semi-arid climate—Israel as a case study. *International Journal of Life Cycle Assessment* 25(6): 1133–1144.

Ministry of Construction and Housing. 2019. *Pricing of insulation methods - industrial construction in Israel (תמחור שיטות בידוד - בנייה מתועשת בישראל).*

Ministry of Environmental Protection. 2021. *A prosperous economy in a sustainable environment - an economic vision document and a roadmap for the years 2020-2030(כלכלה משגשגת בסביבה מקיימת - מסמך חזון משקי ומפת דרכים לשנים 2020-2030).* October. https://www.gov.il/BlobFolder/reports/vision_document/he/sustainability_economy_vision-document.pdf.

Nesher Israel Cement Enterprises Ltd. 2014. *Environmental Product declaration Portland cement-cem II 42.5 n/B-ll*. http://www.assifstrategies.com/.

Notteboom, T. and P. Cariou. 2009. Fuel surcharge practices of container shipping lines: Is it about cost recovery or revenue making? *The Geography of Transport Systems*. https://transportgeography.org/contents/chapter4/transportation-and-energy/fuel-consumption-containerships/.

One Click LCA. 2021a. Reinforcement steel (rebar), generic, 0% recycled content (only virgin materials), A615. https://oneclicklcaapp.com/app/sec/main/list.

One Click LCA. 2021b. Reinforcement steel (rebar), generic, 60% recycled content, A615. https://oneclicklcaapp.com/app/sec/main/list.

One Click LCA. 2021c. Reinforcement steel (rebar), generic, 90% recycled content, A615. https://oneclicklcaapp.com/app/sec/main/list.

Pearlmutter, D., I.A. Meir, and N. Huberman-Meraiot. 2013. *The Embodied Energy of Building Materials in Israel: Development of a National Database Final Report*. http://ilgbcatalog.org/wp-content/uploads/2016/07/Final-report-The-Embodied-Energy-of-Building-Materials-in-Israel.pdf.

Sea-Disrances.ORG. 2021. SEA-DISTANCES.ORG - Distances. https://sea-distances.org/.

Stora Enso. 2020. *Environmental Product Declaration CLT (Cross Laminated Timber)*. www.environdec.com.

The National Economic Council. 2016. Future housing demand in Israel 2016-2040 (צרכי הדיור העתידיים בישראל 2016-2040): 2037–2035.

The Norwegian EPD Foundation and Pretec Norge AS. 2021. *Rebar bolt - HRB500E Pc-Coat - Environmental Product Decleration no. NEPD-2704-1407-EN*. May 3. www.epd-norge.no.

Wu, P., B. Xia, and X. Zhao. 2014. The importance of use and end-of-life phases to the life cycle greenhouse gas (GHG) emissions of concrete – A review. *Renewable and Sustainable Energy Reviews* 37: 360–369.

Zabalza Bribián, I., A. Valero Capilla, and A. Aranda Usón. 2011. Life cycle assessment of building materials: Comparative analysis of energy and environmental impacts and evaluation of the eco-efficiency improvement potential. *Building and Environment* 46(5): 1133–1140.

Zhutovsky, S. and A. Shishkin. 2020. *Recycling of concrete waste for the production of clinker for Portland cement (מחזור פסולת בטון לצרכי ייצור קלינקר של צמנט פורטלנד).* Haifa. https://www.gov.il/BlobFolder/reports/pesolet-beton/he/documents_pesolet-beton.pdf.
